# Personal exposures to fine particulate matter and carbon monoxide in relation to cooking activities in rural Malawi

**DOI:** 10.12688/wellcomeopenres.18050.1

**Published:** 2022-10-11

**Authors:** Sepeedeh Saleh, Henry Sambakunsi, Debora Makina, Martha Chinouya, Moses Kumwenda, James Chirombo, Sean Semple, Kevin Mortimer, Jamie Rylance

**Affiliations:** 1 Liverpool School of Tropical Medicine, LIVERPOOL, L3 5QA, UK; 2 Malawi Liverpool Wellcome Trust Clinical Research Programme, Blantyre, P.O. Box 30096, Malawi; 3 University of Stirling, Stirling, FK9 4LA, UK; 4 Department of Medicine, University of Cambridge, Cambridge, UK

**Keywords:** air pollution; particulate matter; carbon monoxide; exposure; monitoring; cooking

## Abstract

**Background: **Air pollution is a major environmental risk factor for cardiorespiratory disease. Exposures to household air pollution from cooking and other activities, are particularly high in Southern Africa. Following an extended period of participant observation in a village in Malawi, we aimed to assess individuals’ exposures to fine particulate matter (PM _2.5_) and carbon monoxide (CO) and to investigate the different sources of exposure, including different cooking methods.

**Methods: **Adult residents of a village in Malawi wore personal PM _2.5_ and CO monitors for 24-48 hours, sampling every 1 (CO) or 2 minutes (PM _2.5_). Subsequent in-person interviews recorded potential exposure details over the time periods. We present means and interquartile ranges for overall exposures and summaries stratified by time and activity (exposure). We employed multivariate regression to further explore these characteristics, and Spearman rank correlation to examine the relationship between paired PM _2.5_ and CO exposures.

**Results** **: **Twenty participants (17 female; median age 40 years, IQR: 37–56) provided 831 hours of paired PM _2.5_ and CO data. Concentrations of PM _2.5_ during combustion activity, usually cooking, far exceeded background levels (no combustion activity): 97.9μg/m ^3^ (IQR: 22.9–482.0), vs 7.6μg/m ^3^, IQR: 2.5–20.6 respectively. Background PM _2.5_ concentrations were higher during daytime hours (11.7μg/m ^3^ [IQR: 5.2–30.0] vs 3.3μg/m ^3^ at night [IQR: 0.7–8.2]). Highest exposures were influenced by cooking location but associated with charcoal use (for CO) and firewood on a three-stone fire (for PM _2.5_). Cooking-related exposures were higher in more ventilated places, such as outside the household or on a walled veranda, than during indoor cooking.

**Conclusions** **: **The study demonstrates the value of combining personal PM _2.5_ exposure data with detailed contextual information for providing deeper insights into pollution sources and influences. The finding of similar/lower exposures during cooking in seemingly less-ventilated places should prompt a re-evaluation of proposed clean air interventions in these settings.

## Background

Air pollution is the fourth leading risk factor for premature mortality worldwide ^1^. It is estimated to have contributed to 6.67 million deaths in 2019, largely through respiratory and cardiovascular pathology, with the highest risks occurring in low- and middle-income countries (LMICs) ^1, 2^. Across sub-Saharan Africa particularly, poor air quality is a persisting issue, with little of the improvements sometimes seen in more affluent regions ^2, 3^. Household air pollution, from cooking, heating, and lighting, accounts for a large proportion of the deaths attributable to air pollution, particularly in low-income countries in sub-Saharan Africa ^1^; it also contributes to ambient air pollution. In Malawi, where air pollution remains a leading risk factor for morbidity and mortality ^4^, exposure to fine particulate matter (PM _2.5_), defined as particles of diameter <2.5 µm, from household sources, was responsible for an estimated 12,400 deaths in 2019 ^5^. Other common air pollution sources in Malawi include pollution from vehicles and burning of farmland and brick ovens ^6– 8^.

In Malawi and similar settings, PM _2.5_ and carbon monoxide (CO) exposures relate strongly to cooking ^9– 11^ and far exceed internationally agreed cut-offs ^12^. This suggests that cleaner cooking devices might be beneficial ^13– 17^, although provision of these in intervention trials have not significantly improved health endpoints ^18, 19^. Data on additional non–cooking-related sources of air pollution are available, but specific source apportionment in the context of overall daily exposures is uncommon ^14, 20, 21^.

In a recent report from Malawi, we drew insights from in-depth participant observation to inform the design of a monitoring study, providing contextual observational data of cooking behaviour ^8^. Participants’ mobility around the household area, even during cooking episodes, means that stationary monitoring inaccurately reflects personal exposure ^17^. Importantly, individuals within a household use varying sites for cooking, and different fuels and stoves, even within a 24-hour period. More detailed data on cooking-related and additional exposure sources are required to better understand where and to what extent exposures are happening and, therefore, the potential effects of exposure-reduction interventions ^22^. We set out to fill this evidence gap through concurrent personal PM _2.5_ and CO exposure monitoring, coupled with detailed time-activity data to explore the influence of cooking and of individual cooking characteristics, such place, fuel, and device use. This allows us to develop a more granular model of air pollution exposures. We also examined the relationship between paired PM _2.5_ and CO exposures, adding to the existing evidence on correlates of air quality in this context.

## Methods

### Ethical considerations

The study was approved and sponsored by the LSTM Research Ethics Committee (19-007). In-country ethical approval was granted by the College of Medicine Research Ethics Committee (COMREC) in Blantyre (P.06/19/2600). Written informed consent processes were completed for all participants involved in air quality monitoring. Further information around ethical aspects of the study has been published separately ^23^.

### Study design

This study was nested within a larger ethnographic study which incorporated extended participant observation concurrent personal PM _2.5_ and CO exposure measurement in a Malawian village ^8^. Household-based participant observations in and around the village took place between July 2019 and January 2020 (during the hot season and part of the cooler rainy season in Malawi), with observations and preliminary quantitative data collected from researchers through proxy exposure sampling informing the sampling design.

**Figure 1.**
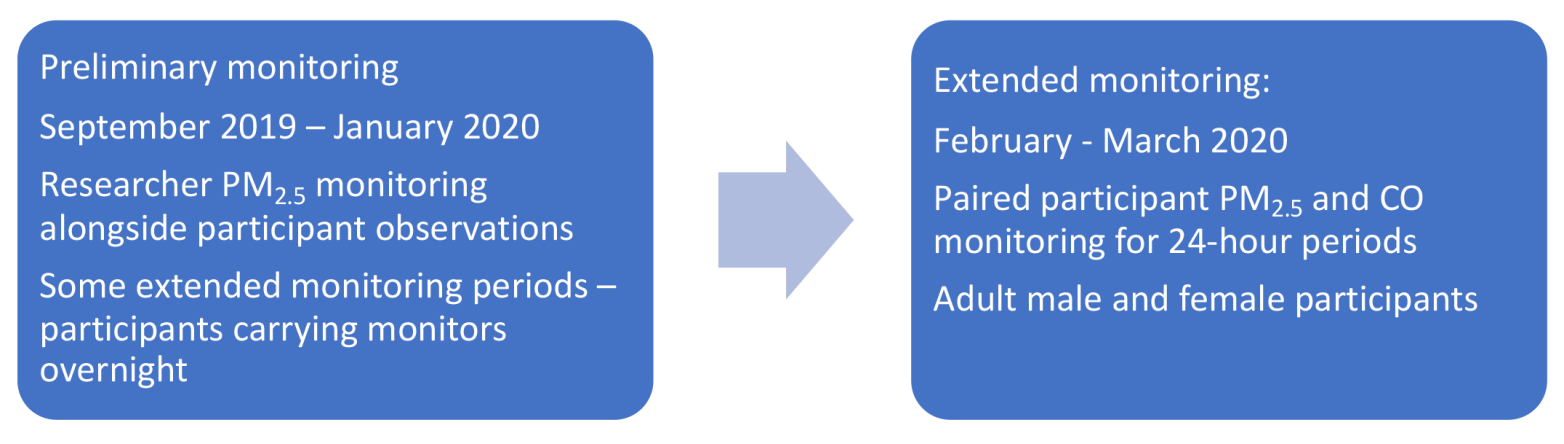
Phases of air quality monitoring.

Summary measures from the preliminary phase have been reported separately ^8^. Definitive exposure data reported in this paper reflect results of 48-hour personal monitoring in a cohort of village participants between January and March 2020 ( Figure 1) (Extended data ^24^).

### Study setting

Participants lived in a rural village of approximately 840, comprising 722 adults: 380 men and 342 women (population data from local health surveillance assistant, personal communication, 30 September 2021). During daylight hours, the adult population present in the village was largely female, as many men travelled to neighbouring areas seeking employment. The village was 12 km from Blantyre, the commercial capital of Malawi, and approximately 2 km from nearest tarmac single-carriageway road. Much of the area was not accessible by any type of road. Village life focussed around subsistence farming, reflecting the lifestyle seen across most rural communities in the country ^25^. Prior participant observation of cooking patterns in the village demonstrated that three-stone fires were habitually used in almost all households, with some individuals also using charcoal and firewood stoves. Individuals’ stove and fuel use and place of cooking often varied by weather, food cooked (or other stove activity, such as bathwater warming), and occasion. Further details of the setting, including common cooking modalities, have been published separately ^8^.

### Participants

Adult male and female residents (>18 years of age) spending at least 6 days of the week in and around the village were invited to participate. Details of recruitment and related study approaches are discussed separately ^23^. Only participants giving written informed consent were included. People aged 18 or under, or unable to provide informed consent, were excluded.

### Data collection

***PM _2.5 _and CO measurement.*** Participants each spent 48 hours carrying two personal air quality monitors in waist bags specifically designed for this study. PurpleAir PA-II-SD laser particle counting devices (PurpleAir, Draper, UT, USA) with 20-Ah portable power banks (Anker Innovations, Changsha, China), previously employed in a number of African settings ^26, 27^, logged PM _2.5 _concentrations at 2-minute intervals. LASCAR EL-USB-CO devices (Lascar Electronics, Erie, PA, USA) logged CO concentrations every minute. Each PurpleAir monitor was positioned on a large hole in the base of the bag, and the CO data logger protruded from a zip pocket.

***Activity data.*** At the end of each 24-hour monitoring period, potential exposures were identified through an in-person review of PM _2.5 _traces created from PurpleAir data using a line graph in Excel, Version 16.63 (Microsoft, Redmond, WA, USA) ^28^, viewed on a laptop screen by the participant and a researcher together. Information on potential exposures were gathered at this point, guided by participant recall (around cooking periods each day, for instance), together with visible peaks on traces. Data on potential exposures covered the following key areas, informed by observations during the preceding fieldwork period and preliminary monitoring:

1 Combustion source, including:

- Cooking/bathwater warming/other household fires
- Farming-related exposures
- Traffic exposure
- Other

2 For cooking-related exposures, additional data were gathered on:

a) Place of cooking:
  - ‘Indoors’ – either inside the household or in an enclosed kitchen
  - Kitchen with no roof
  - Walled *khonde* (veranda)
  - *Khonde* with no walls
  - Outdoors (in yard area)
b) Device used for cooking:
  - Three-stone fire
  - Charcoal cookstove
  - Firewood cookstove
c) Fuel used for cooking:
  - Firewood
  - Charcoal
  - Other

### Data management and statistical analysis

For PM _2.5_, ‘CF=1’ values were selected, on expert advice, in view of key environmental features. The PurpleAir PM _2.5_ monitors each have two separate sensors. Data from these was managed by checking, for each trace, that readings from both sensors were in agreement throughout, before using an average of the two values for the analysis. Times for these devices were set through connection to the internet, with regular reconnection ensuring no significant drift. Each 2-minute PM _2.5_ concentration was paired with CO concentration, and with activity data, and these data used for subsequent analyses.

Matching time-activity data generated through interviews were used to indicate which periods on each trace represented ‘activity’ (when there was an identified exposure source present), with the remainder of the time points constituting ‘background’ exposures (no identified source of combustion present). More detailed activity data also allowed analysis by cooking details (device, fuel, and place of cooking).

Medians and interquartile ranges (IQRs) for PM _2.5_ and CO during ‘activity’ periods were calculated and compared with the remainder, identified as ‘background’ exposures, across the full dataset. Medians and IQRs were also calculated for daytime background exposures (05:00 h to 22:00 h) and compared with background exposures through the night (22:00 h to 05:00 h). Selection of these time categories was informed by the previous ethnographic work in the village. Medians and IQRs were preferred over means throughout the analysis in view of the skewed nature of the exposure data and in line with other work in the area ^11, 21, 29^.

The medians and IQRs of all datapoints across the dataset during cooking were compared with those associated with ‘no activity', and summary measures were similarly used to compare various cooking characteristics (cooking device, fuel, and place of cooking). For boxplots, CO +1 values were used before log transformation to allow for transformation of zero values.

Multivariate regression models were employed to explore the effects of these cooking characteristics in greater detail, while also acknowledging autocorrelation between datapoints from the same participant over time (hence the use of mixed models).

Correlation between paired PM _2.5_ and CO exposures was analysed both visually using a scatter plot and through the calculation of a Spearman rank correlation coefficient. All data were analysed using R (R Foundation for Statistical Computing, 2020, Vienna, Austria) (RRID:SCR_001905) ^30^, and figures were created using the package ggplot2 (RRID:SCR_014601) ^31^. Linear regression was done using the lme4 package (RRID:SCR_015654) ^32^ and outputs created using the Stargazer package ^33^.

## Results

**Figure 2.**
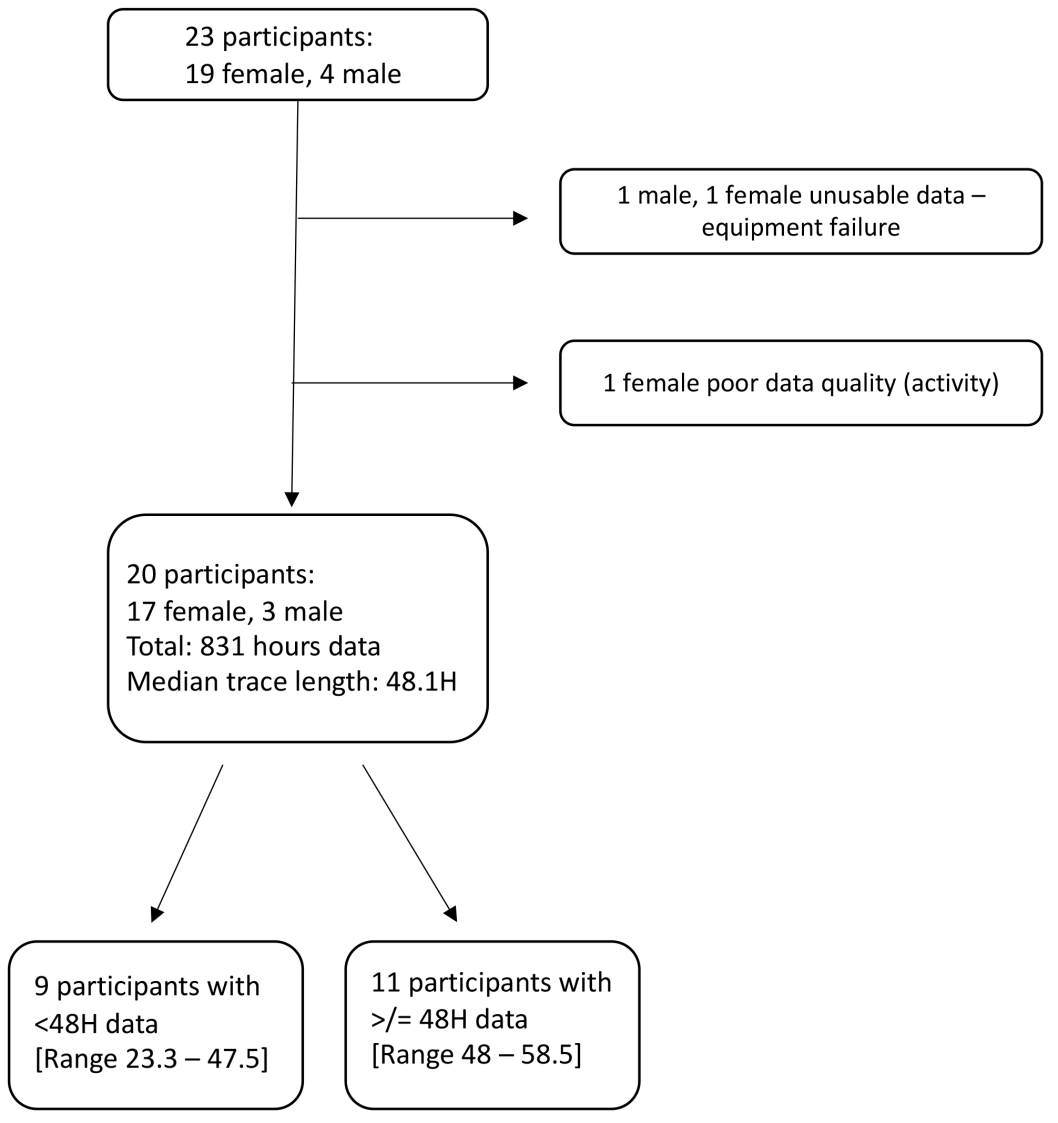
Flow chart depicting participants included and excluded, with data on duration of monitoring.

The extended dataset included a total of 831 hours of paired PM _2.5_ and CO exposure data from 20 participants, all of which was included in the analysis ( Figure 2). 11 of these 20 participants had two full contiguous 24-hour traces amounting to more than 48 hours of monitoring. Shorter samples were due to battery faults, but there were no individual or sporadic missing values within the existing data traces.

**Figure 3.**
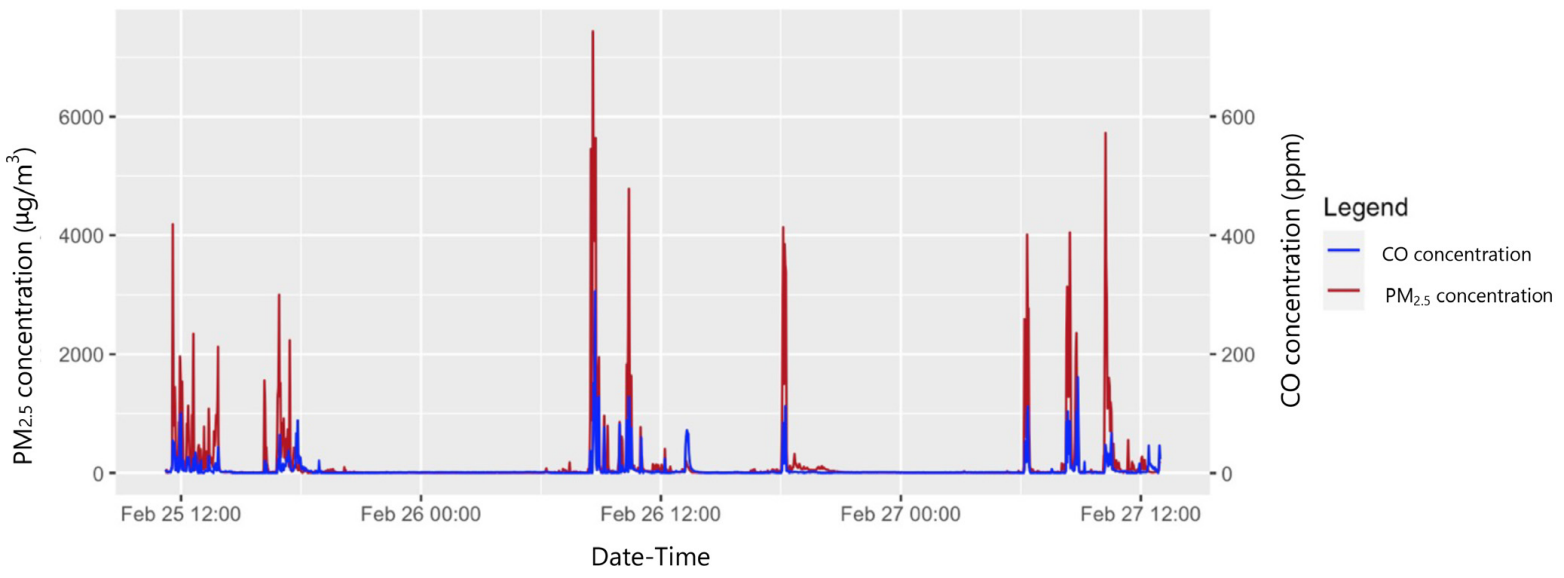
Variation in PM _2.5_ and CO concentrations over a 48-hour time-period in a sample participant.

Both PM _2.5_ and CO traces showed a ‘baseline + peak’ pattern, with echoing patterns in paired traces ( Figure 3).

Testing for normality using the Shapiro–Wilk test revealed the data to be highly skewed, with a left skew representing lower PM _2.5_ concentrations (in the absence of combustion activity), and a long tail representing PM _2.5_ concentrations reaching >1,000 μg/m ^3^ during cooking activity.

### Activity-related and background exposures

‘Peaks’, or periods of ‘activity’ (where there was an identified source of combustion) represented 23% of the overall recording period. Median PM _2.5_ exposure during these activity periods was 97.9 μg/m ^3^ (IQR: 22.9–482.0), whereas median PM _2.5 _background exposure concentrations (at times of no identified combustion sources) were 7.6 μg/m ^3^ (IQR: 2.5–20.6). This comparison is shown in the box plots (( Figure 4a), which also depict the wide dispersal of values, which often reached above 1,000 μg/m ^3^ during periods of ‘activity’. Median carbon monoxide exposure during periods of identified activity was 4 ppm (IQR: 1–12), compared with median background exposures of 0 ppm Figure 4b).

**Figures 4a & b.**
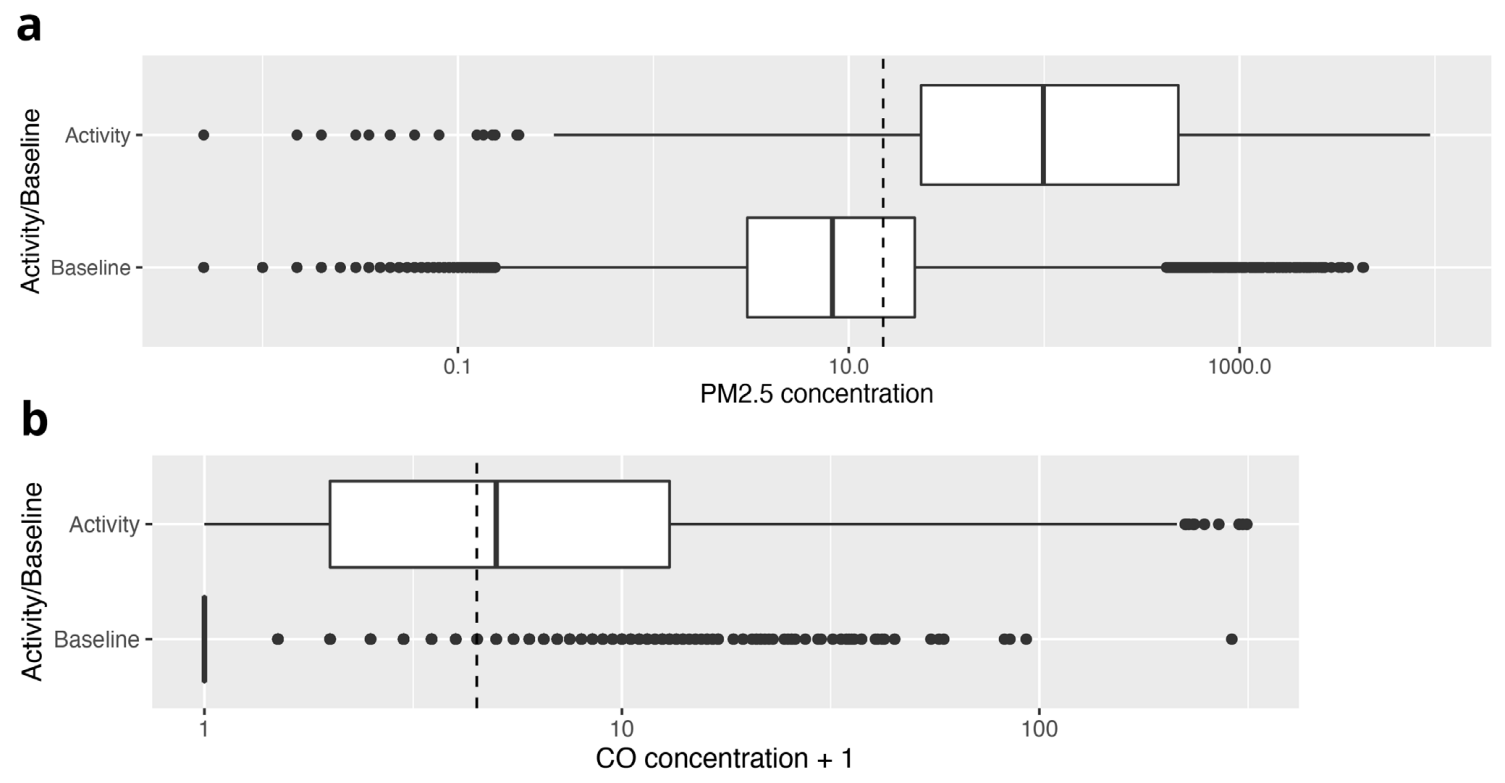
Box plots depicting median PM _2.5_ and CO exposures during periods of combustion activity and at baseline (background exposures) across the dataset, with PM _2.5_/CO concentrations plotted on a log scale. Dotted lines indicate WHO-recommended 24-hour upper limits (PM _2.5_ concentration 15 μg/m ^3^; CO concentration 4 mg/m ^3^ = 3.492 ppm) ^12^

Of the total ‘activity’ time period, 86% represented cooking or a related activity in the household (including starting a cooking fire and use of this fire—or cookstove—for warming bathwater and warming oneself). Other exposure sources captured in the dataset included burning grass at the farm, proximity to a minibus, soldering of a radio, and an identified cooking fire in a neighbouring household.

When ‘no activity’ periods were stratified by diurnal period, there were 399 hours of ‘no activity’ data during the day, compared with 237 hours at night. Median ambient PM _2.5 _exposures were higher in the day than the night ( Figure 4c): 11.7 μg/m ^3^ [IQR: 5.2–30.0] and 3.3 μg/m ^3^ [IQR: 0.7–8.2] respectively.

**Figures 4c & d.**
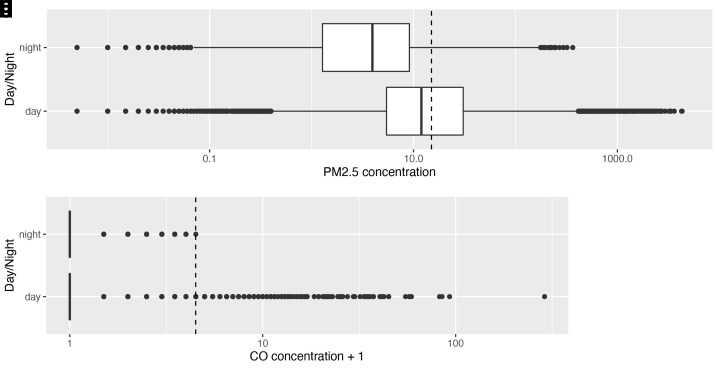
Box plot depicting background median PM _2.5_ and CO exposures (where no identified combustion activity), during daytime and night-time hours, with PM _2.5_/CO concentrations plotted on a log scale. Dotted lines indicate WHO-recommended 24-hour upper limits (PM _2.5_ concentration 15 μg/m ^3^; CO concentration 4 mg/m ^3^ = 3.492 ppm) ^12^

Male and female exposures were not compared because of the small number of male participants involved in this study.

### Cooking characteristics

Of all identified cooking time, 80% involved the use of a three-stone fire. The remainder of the cooking time involved either charcoal or firewood cookstoves (10% and 9%, respectively). Indoor cooking was most common (60% of total cooking time, of which 82% was in a closed kitchen, and the remainder in a house). Less commonly, cooking was done on walled verandas (24% of all cooking time), outside (11%), or on open verandas (no walls). Only one participant cooked in a kitchen with no roof (2% of total cooking time).

**Figures 5a & b.**
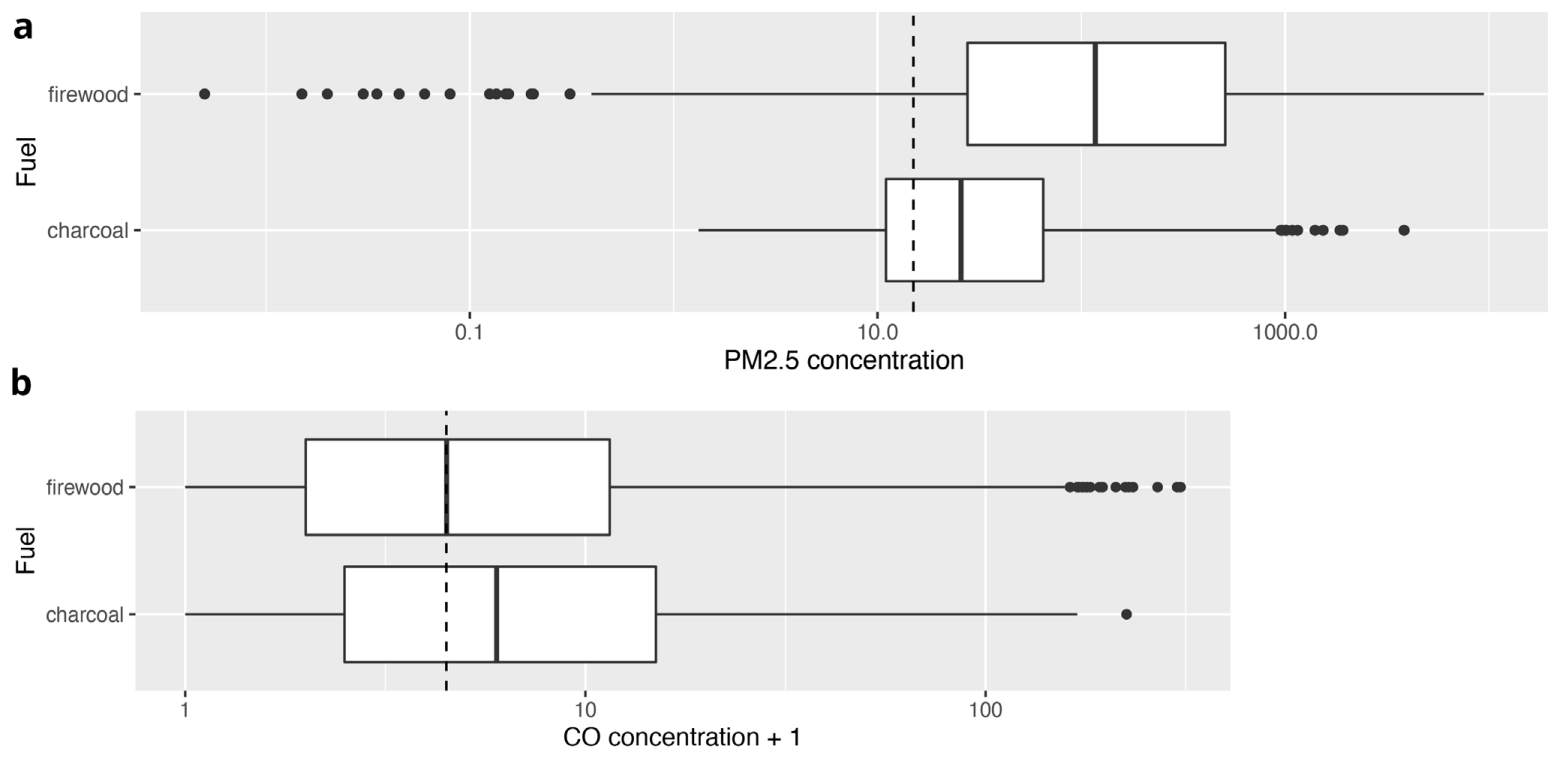
Box plot depicting median cooking related PM _2.5_ and CO exposures during cooking episodes using firewood compared with those using charcoal, with PM _2.5_/CO concentrations plotted on a log scale. Dotted lines indicate WHO-recommended 24-hour upper limits (PM _2.5_ concentration 15 μg/m ^3^; CO concentration 4 mg/m ^3^ = 3.492 ppm) ^12^

Univariate analysis suggested that use of firewood was associated with higher PM _2.5_ exposures than charcoal (median 115.0 μg/m ^3^ [IQR: 26.7–506.0] *versus* median 25.7 μg/m ^3^ [IQR: 11.0–65.0] for charcoal). In contrast, CO exposures were slightly lower during cooking periods using firewood compared with charcoal (median 3.5 ppm [IQR: 1.0–10.0] *versus* median 5.0 ppm [IQR: 1.5–14.0]). These differences are shown in Figures 5a & b.

Use of three-stone fires was associated with higher PM _2.5_ exposures than either firewood or charcoal cookstoves (median 127.0 μg/m ^3^ [IQR: 30.7–535.0]; median 39.5 μg/m ^3^ [IQR: 9.8–221.0]; median 26.7 μg/m ^3^ [IQR: 11.3–68.0], respectively). This again contrasted with CO concentrations, which were lower during cooking episodes using firewood stoves than with either three-stone fires or charcoal stoves (median 1.0 ppm [IQR: 0.0–3.0]; median 4.0 ppm [IQR: 1.5–11.5]; median 5.0 ppm [IQR: 1.5–14.0], respectively).

All cooking episodes could be represented by one of three combinations:

1 Firewood on a three-stone fire
2 Firewood on a firewood cookstove
3 Charcoal on a charcoal cookstove

Fuel and stove were, therefore, combined into a single ‘fuel_stove’ categorical variable for the purposes of the regression model. The full model thus includes ‘fuel_stove’ and ‘place of cooking’ as fixed effects and participant number as a random effect (in recognition of the likely individual/household-level determinants involved). The dependent variable was log normalised using (log _10_(1+[PM _2.5_])) to allow treatment of zero values. Results of regression analyses presented here only relate to the PM _2.5_ outcome. Results of the regression model using CO as a dependent variable have been included in the Extended data ^24^.


(log⁡10(1+[PM2.5]))∼'place'+'fuel_stove'+(1|'participant'))

An initial mixed model examining fuel_stove alone (with ‘participant’ as a random effect) indicated that use of firewood—either on a three-stone fire, or on a firewood cookstove—predicted higher PM _2.5_ exposure compared with use of charcoal on a charcoal stove. The increase in exposure was greater for firewood on a three-stone fire (estimate = 1.25, error = 0.095, *P*<0.01) than for firewood on a firewood cookstove (estimate = 0.25, error = 0.14, *P*<0.1).

A similar mixed model using ‘place of cooking’ alone indicated that—compared with cooking indoors—cooking in a kitchen with no roof, walled veranda, or outside the household were all significantly associated with higher exposures ( *P*<0.01 in all three cases). Cooking in an unwalled veranda in this model appeared to be associated with higher exposures ( *P*<0.01). Both models indicated that inter-participant variation was less than variation due to other factors.

Compared with the fuel_stove–only model, adding place of cooking (to give the full model) significantly improved the prediction of PM _2.5_ exposures (χ ^2 ^(4) =23.7, ANOVA *P*=0.001). This model affirmed the significance of fuel_stove in shaping exposures, with wood on a three-stone fire significantly associated with higher exposures than charcoal used on a charcoal stove (estimate = 1.12, error = 0.11, *P*<0.01); firewood on a firewood stove was, in this model, not associated with significantly different exposures than charcoal. In the full model, compared with cooking indoors, cooking in a walled veranda or outside the household were associated with significantly higher personal exposures (Extended data ^24^, Table S1). Cooking taking place in a kitchen with no roof and in an unwalled veranda were not associated with any significant differences.

### Correlation between PM _2.5_ and CO concentrations

On visual inspection of a contour plot with an overlaid line of best fit ( Figure 6a), there appeared to be a correlation between PM _2.5 _and CO concentrations across the whole dataset. The Spearman rank correlation coefficient (r _s_) was 0.50 ( *P*<0.001), indicating a moderate correlation between PM _2.5_ and CO concentrations overall.

**Figure 6a.**
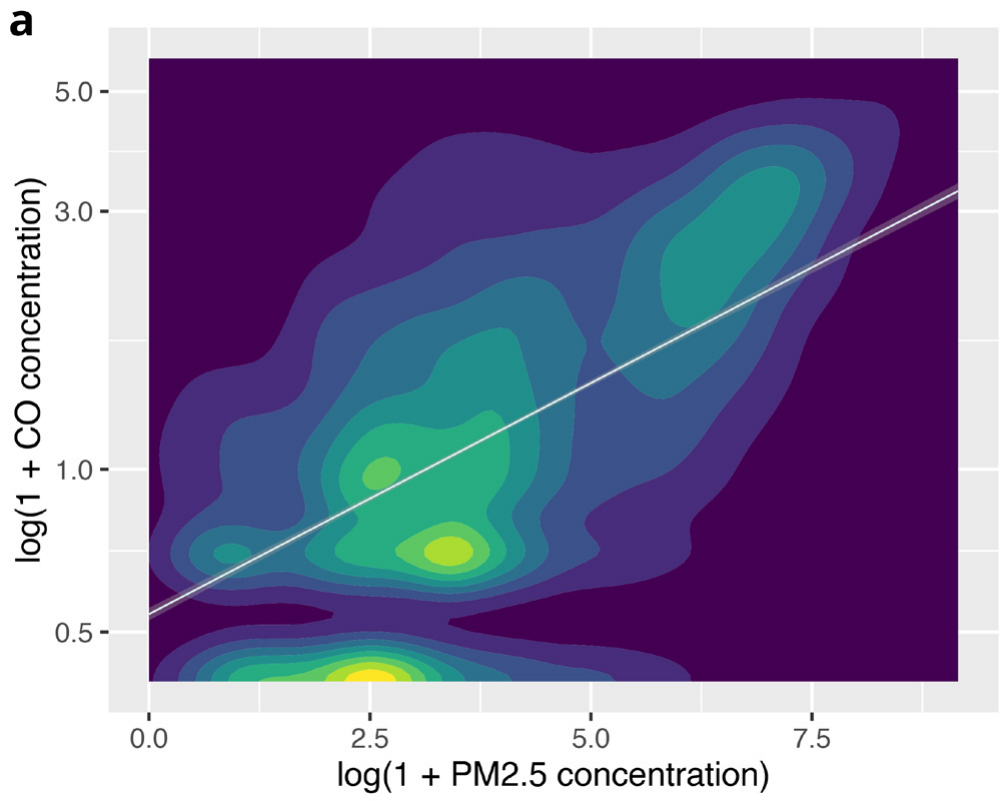
Contour plot illustrating the relationship between PM _2.5_ and CO across the complete dataset, using log(1+CO) and log(1+ PM _2.5_).

The apparent clustering in this graphic was explored using separate plots for ‘cooking’ and ‘background’ periods ( Figures 6b & c). Analysis of correlation in these subgroups found a stronger relationship during cooking activity (r ^2^=0.42) compared with background periods (r ^2^=0.22).

**Figures 6b & c.**
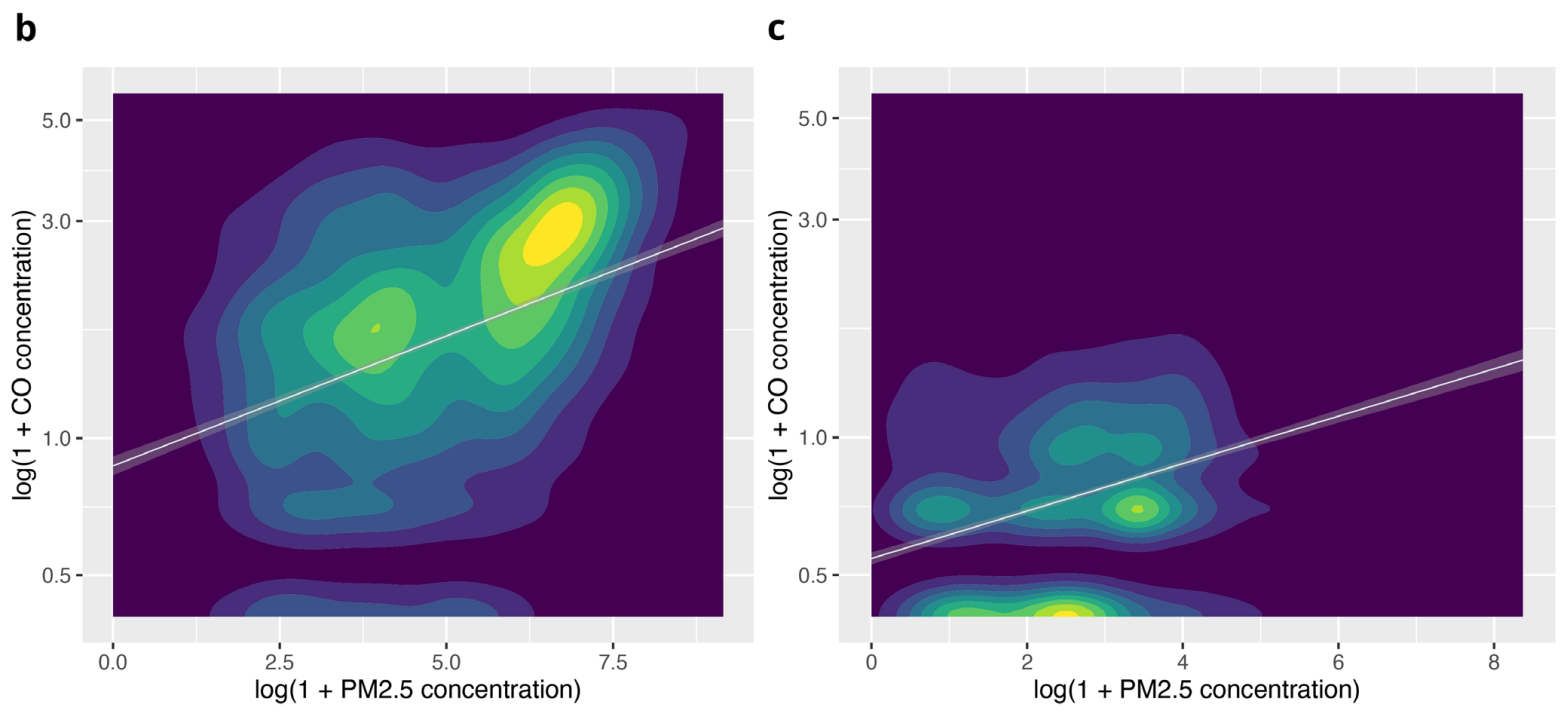
Contour plots illustrating the relationship between PM _2.5_ and CO during cooking activity, and background periods (no identified combustion activity), using log(1+CO) and log(1+ PM _2.5_).

## Discussion

Our personal monitoring results, coupled with in-depth data around daily exposures, demonstrated the primacy of cooking in individuals’ exposure landscapes in Malawi. Median PM _2.5_ and CO exposures were significantly higher during activity (usually representing cooking) than background exposures, in the absence of identified combustion activity. Analysis of paired cooking data revealed the use of wood on a three-stone fire to be significantly associated with higher exposures than cooking using charcoal or firewood stoves, and cooking in a walled veranda or outside the household were associated with significantly higher personal exposures than cooking outdoors.

The data indicated that median background PM _2.5_ and CO concentrations—7.6 μg/m ^3^ and 0 ppm for PM _2.5_ and CO, respectively—were below World Health Organization (WHO)-recommended 24-hour levels ^12^ but that cooking episodes frequently exposed participants to extremely high pollutant concentrations (PM _2.5_ often >1,000 μg/m ^3^). High pollutant concentrations have been previously reported in this setting ^9, 34^, but using personal monitoring with paired activity data, we were able to separately analyse background and peak PM _2.5_ concentrations, framing cooking as a key exposure source. This echoes findings from Uganda, Ethiopia, and Ghana ^14, 17^, with further analysis exploring specific factors which shape cooking-related exposures.

The diurnal difference in background PM _2.5_ concentrations reveals the contribution of daily activity across the village to ambient levels. This contrasts with data from more urban LMIC settings, which describe higher pollutant concentrations at night, likely driven by atmospheric changes related to cooling ^35^. While our observations in and around the village revealed a variety of potential contributors to air pollution ( *e.g*., burning farmland, environmental dust), cooking clearly constituted the primary source of exposure for participants in the village environment ^8^. The shared nature of air pollution here demands interventions which can be near-universally adopted in a given geographical area ^36, 37^.

Following an initial period of ethnographic observation for better understanding of the context, personal monitoring paired with fine-grain data on individual cooking episodes, collected after each monitoring period, allowed for analysis of personal cooking exposures by fuel, device, and place of cooking. The association of lower PM _2.5_ concentrations with charcoal cooking reflected community members’ own understandings and echoed findings in the literature ^38^. Small reductions in PM _2.5_ concentrations with use of firewood cookstoves compared with three-stone fires supports the use of these low-cost local stoves, although the health impacts of such modest reductions are unclear ^39, 40^.

Personal PM _2.5_ exposures associated with cooking indoors were found to be lower than exposures associated with cooking outdoors or on walled verandas and no different from exposures encountered while cooking in other structures. While the idea that cooking in apparently better-ventilated places might be associated with similar or higher exposures than cooking in more enclosed spaces initially seems counterintuitive—and counter to the mainstream discourse ^41– 44^—cooking patterns regularly witnessed in the village help explain these effects. We frequently noted that women cooking in smoky kitchens spent time sitting outside or away from the kitchen between visits to tend the fire or the pot, whereas cooking done in a more ‘social’ space, such as a veranda, involved the cook, as well as family and friends, spending extended periods by the fire. In view of the high PM _2.5_ concentrations produced during any cooking activity, periods of physical distancing from the site may plausibly produce similar or more marked reductions in personal exposures than continuous cooking in spaces with a degree of ventilation. Awareness-raising interventions around the harms of ‘smoke’ and support for women to spend less time close to cooking devices could constitute a first step to reducing exposures in the village setting, although structural changes to overcome contextual limitations will be required to achieve sustainable improvements ^45^.

Concurrent measurements revealed a strong association between individual PM _2.5_ and CO exposures at peak concentrations but an absence of this association during background periods. This builds on review-level evidence from a range of global settings indicating inconsistencies in the correlation ^46, 47^. In view of the clinical significance of background concentrations of pollutants, even where peak concentrations are reduced ^12, 48, 49^, our findings indicate weaknesses in the use of CO measurement as a proxy for PM _2.5_ exposure. Our successful use of small, soundless, portable PM _2.5_ monitors establishes their utility in personal exposure monitoring and, in view of the similarities in costs of the two monitors, favours their use for direct PM _2.5_ monitoring, superseding use of proxies.

The current study involved a relatively small number of participants, preventing detailed regression analyses and more precise models. Residual variation in cooking exposures, possibly related to firewood type or moisture content, type of food cooked, or daily weather conditions, was unexplained by the current models. Observations in the village suggested a role for these factors in influencing cooking related PM _2.5_ concentrations, in keeping with evidence from other studies ^50– 52^, but difficulties in quantification and sample size limitations precluded their incorporation in the analysis.

The retrospective reviewing, with participants, of traces on laptop screens to determine exposure periods could potentially have introduced recall bias in exposure categorisation. Combustion activities tended to create clear exposure peaks ( Figure 3), but timing inaccuracies could lead to misclassification of datapoints around the start and end of activities. This system was used because while village residents tended to split their days broadly into ‘morning’/‘afternoon’/‘evening’ (with lunch usually consumed at around 12 o’clock), they were otherwise generally unaware of the time and did not use watches or clocks at all. Together with the predominance of spoken (over written) communication, this precluded the use of self-completed activity diaries, for example.

The use of medians rather than means in this study—in keeping with other similar studies ^11, 21, 53^—reduced the effects of potential exposure misclassification, and whilst still constituting an inherent risk in the study design, this is unlikely to have significantly impacted the key study findings around diurnal variation or cooking characteristics for example.

Further study limitations include a relatively short study period (excluding certain seasonal variations, such as changes in fuel use) and the occurrence of very high PM _2.5_ values (>250 µg/m ^3^) during cooking-related peaks, lying outwith the calibration range of the instruments ^26^. This highlights the need for gravimetric calibration of the monitors in rural sub-Saharan African settings but does not change the direction of inference of the current results.

## Conclusions

High cooking-related PM _2.5_ and CO concentrations in this study and a raised background level during the day compared with night signal the need for accessible, population-wide approaches to achieve clinically meaningful exposure reductions. The study demonstrated the feasibility of direct PM _2.5_ monitoring using personal devices, which is important, given our finding of poor PM _2.5_–CO correlation during background (non-activity) periods. The finding of lower or similar exposures during cooking in less-ventilated places outlines the value of our personal, activity-matched monitoring approach, together with detailed participant observations in the setting. This gives added value to exposure assessment and consequent decisions surrounding interventions and their evaluation.

## Data availability

### Underlying data

Harvard Dataverse: Underlying data and code for “Personal exposures to fine particulate matter and carbon monoxide in relation to cooking activities in rural Malawi”. https://doi.org/10.7910/DVN/7A0XIS ^24^

The project contains the following underlying data:

- Original data - anonymised
- R code for analysis

### Extended data

Harvard Dataverse: Extended data for “Personal exposures to fine particulate matter and carbon monoxide in relation to cooking activities in rural Malawi”. https://doi.org/10.7910/DVN/7A0XIS ^24^

 This project contains the following extended data:

- Extended data - ‘Personal exposures to fine particulate matter and carbon monoxide in relation to cooking activities in rural Malawi’

Data are available under the terms of the Creative Commons Zero "No rights reserved" data waiver (CC0 1.0 Public domain dedication).
