# Supplementary material for: Personal exposures to fine particulate matter and carbon monoxide in relation to cooking activities in rural Malawi
Source: Wellcome Open Res. 2023 Feb 21;7:251. Originally published 2022 Oct 11. [Version 2] doi: 10.12688/wellcomeopenres.18050.2 (PMC9975423; doi:10.12688/wellcomeopenres.18050.2)
Supplement: Peer review history

# Peer review: Reviewer response for version 1

**DOI:** 10.21956/wellcomeopenres.20017.r53467

**Published:** 2022-12-14

**Authors:** Suzanne E. Bartington

**Affiliations:** 1 Institute of Applied Health Research, University of Birmingham, Birmingham, UK

This is a well conducted study, investigating cooking-related exposure to PM _2.5_ and CO among a sample of adult residents living in biomass fuel households in rural Malawi. Importantly, this study links contextual information and time-activity behaviour with primary paired measurements of PM _2.5_ and CO thereby demonstrating the value of combining this information to understand personal exposure profiles; of relevance for epidemiological and health impact assessment studies. 

 Although the study was conducted using a small convenience sample (20 participants) it includes 831 hours of data collection for the respective individuals. The data gaps experienced are typical for primary data collection in this context, with just 11 or 20 participants providing full 24-hour traces. This learning is important for future logistical considerations for personal exposure assessment studies in LMIC settings, where reliance upon battery/powerbank powered devices is typical. Further methodological detail would be welcome for the approaches used to calibrate sensors, both pre-and post- field deployment. 

 Clear differences were observed for PM _2.5_ and CO concentrations during peak periods of activity, compared to background concentrations, consistent with previous literature in domestic biomass combustion contexts (e.g. Bartington *et al. *(2017) ^1^). Similarly, the finding of higher exposures associated with cooking using three-stone fires when compared to wood or charcoal stoves is consistent with previous investigations. However, some further detail regarding the fuel used for the three-stone fires would be beneficial - including if this is a mix of sources (e.g. straw/dung) and also information as to which fuel is used to light the stove. The findings also suggest an important contribution of daily activities in this context to ambient pollutant levels - of relevant to future research in rural LMIC settings. 

 A key limitation of the study was reliance upon recall for activity data and it is unclear how this was validated against objective measures. However, given the ethnographic study context, the involvement of participants is also relevant providing an opportunity for them to explore potential exposures together with a researcher. This approach may also yield a greater depth of understanding than could be achieved by alternative methods for recording time-activity data, which may omit relevant information. Further limitations are discussed in detail by the authors, including a relative short study period and concentrations which exceed the calibration range of instrumentation. Reproducibility is also unclear given study findings are based upon single monitoring campaign. However, overall this study demonstrates utility of this approach for personal exposure assessment in this rural context, of value to inform future studies.

Is the work clearly and accurately presented and does it cite the current literature?

Yes

If applicable, is the statistical analysis and its interpretation appropriate?

Yes

Are all the source data underlying the results available to ensure full reproducibility?

Yes

Is the study design appropriate and is the work technically sound?

Yes

Are the conclusions drawn adequately supported by the results?

Partly

Are sufficient details of methods and analysis provided to allow replication by others?

Yes

Reviewer Expertise:

Environmental epidemiology, public health

I confirm that I have read this submission and believe that I have an appropriate level of expertise to confirm that it is of an acceptable scientific standard.

## References

[1] : Patterns of domestic exposure to carbon monoxide and particulate matter in households using biomass fuel in Janakpur, Nepal. *Environ Pollut* .2017;220(Pt A) : 10.1016/j.envpol.2016.08.074 38-45 10.1016/j.envpol.2016.08.074 27707597PMC5157800

# Response

**Published:** 2023-02-06

**Authors:** Sepeedeh Saleh

**Affiliations:** Liverpool School of Tropical Medicine, UK

We thank the reviewer for this thoughtful review which raises some useful points for consideration. The following are responses to individual points in the review and we are submitting an edited version of the paper with relevant changes in response to reviewer considerations.

 Regarding calibration of the devices, this has been done by others - as cited in the paper - in settings including African environments similar to that in which the current work is set. Our own planned calibration was prevented, however, by problems relating to the gravimetric monitors we planned to use. This is, as mentioned, a limitation of the current study and we highlight our recommendation that further, more extensive monitoring work in this setting should incorporate formal, site-specific calibration as a first step.

 In response to the queries surrounding fuels used, three stone fires used in the village were great majority of the time, used with collected firewood. Acknowledging this, we devised the three categories of ‘fuel_stove’ (Firewood on a three-stone fire; Firewood on a firewood cookstove; Charcoal on a charcoal cookstove) for the analytic regression model, which considered fuel and device in simple combined categories. We agree that there were in reality subtleties in cooking fuel which were not fully captured in this model (for example when additional substances were added to the fire or used for the initial lighting process, or when firewood was damp) but this categorisation was broadly characteristic of the main cooking practices and represented a clear and measurable model. The use of maize cobs and stalks was seen during the extended participant observation, but only during specific periods (around harvesting time), which were outside of the observation period for this study. This is indicated in the discussion around ‘seasonal variations’. Dung was not generally used as a fuel in this setting.

 We agree fully with the reviewer's points around daily activities and contributions in the village and refer the reviewer to related comments of the first reviewer around these questions, and to our responses.

 Finally, we appreciate the reviewer's point around limitations associated with the element of recall in identification of exposures, but highlight the fact that this was carried out on a background of an extended period of in-person participant observation in the village with concurrent personal exposure monitoring (which showed a characteristic baseline + peak pattern). Findings around context and exposure sources – formally reported elsewhere – paved the way for the use of participant recall in combination with reviewing of traces in the current study. We would not advise the use of these methods in isolation without the prior investigations and understanding of the setting.

# Peer review: Reviewer response for version 1

**DOI:** 10.21956/wellcomeopenres.20017.r52744

**Published:** 2022-10-31

**Authors:** Deborah Stanistreet

**Affiliations:** 1 Department of Epidemiology and Public Health Medicine, Royal College of Surgeons in Ireland, Dublin, Ireland

This paper is well constructed and organised and explores an extremely important issue; that of household air pollution, a major health burden in many low income countries. Around 40% of the global population still do not have access to clean fuels for cooking and the issue is only likely to be compounded by the current energy crisis.

 Using ethnographic observations alongside survey data is a methodology which is underused in the HAP field. This paper makes a powerful case that adding context to survey data aids greatly in data interpretation. In fact, I would argue that mixed methods including observations, should be a standardised methodology in understanding household air pollution in relation to cooking practices. The author makes a good case for this in a previous paper ^1^, (which could also be referenced in relation to this).

 The paper has used standard methods of personal exposure measurement, but as the authors state, personal exposure is a more valid measurement of the impact of exposure on health than those studies that measure levels of pollution close to the stove. In terms of methods, the matching of time activity to the HAP measurements is also a useful demonstration of the importance of context. I think it would be useful to explore other additional activities that may be related to the measurements produced.? (See later comments).

**Results**

 The authors distinguish between a three stone fire, cookstove and also charcoal stove. Could you identify which cookstove(s) was / were being used? This may be significant in that not all ‘improved’ cookstoves have shown demonstrated reductions in PM2.5 under lap conditions, but there is some evidence that the chitetetzo mbaula does reduce HAP and this is the most commonly available ‘improved’ stove in Malawi. e.g. Jagger *et al.* (2017) ^2^

 I would personally be keen to ensure that improved cookstoves are all identified by name so that the reader can assess whether there is any independent evidence that it is more fuel efficient and / or reduces HAP.

 “ *Median PM2.5 exposure during these activity periods was 97.9 µg/m3 (IQR: 22.9–482.0) demonstrating a wide range in terms of level of PM2.5.”*

 Were you able to explore any of the reasons for this? For example, it would be useful to know which cooking practices resulted in exposure to the highest levels of PM2.5. Can you provide any further information about the houses? Any windows / ventilation?

 How homogenous were the cooking practices? In Malawi, Nsima, the staple dish, requires stirring , especially the last ten minutes or so, so the cook is required to stay close to the pot. Conversely, beans require less watching or stirring, but potentially may lead to greater exposure as they require much longer to cook. These factors can also increase exposure.

 Were there any other behavioural practices that could have contributed to greater exposure? E.g. Did you observe any Crouching and blowing air by mouth on burning of wet wood (did anyone dry wood prior to use for instance) . Were there any other behavioural differences such as burning of maize cobs, stalks or burning of plastic on the fire, or use of pot lids which would reduce cooking times? Did some participants maintain the stove for warmth overnight.

 Was lighting a further source of pollution or was it mostly battery lighting? 

 If the authors have any information on these factors, which could potentially explain differences in exposure, these would be useful to include, even within the discussion.

 The finding that cooking on a veranda led to greater exposure is a very interesting one!

 The authors discuss the validity of using CO as a proxy for PM2.5. How common is this? Could you provide a brief discussion? I only found a handful or recent publications on a quick scan and wonder whether 67a, 6b and 6c are of real value to the main message on this study, but I am open to be corrected!

 Finally other issues that might be touched on during the discussion include:

- If / whether cooking practices vary with season?
- Any language issues. Was the spoken language of the community, Chichewa

Overall, an excellent paper, which has huge value in promoting the use of ethnography to provide context when interpreting HAP data.

Is the work clearly and accurately presented and does it cite the current literature?

Yes

If applicable, is the statistical analysis and its interpretation appropriate?

Yes

Are all the source data underlying the results available to ensure full reproducibility?

Yes

Is the study design appropriate and is the work technically sound?

Yes

Are the conclusions drawn adequately supported by the results?

Yes

Are sufficient details of methods and analysis provided to allow replication by others?

Yes

Reviewer Expertise:

Epidemiology, mixed methods, household air pollution

I confirm that I have read this submission and believe that I have an appropriate level of expertise to confirm that it is of an acceptable scientific standard.

## References

[1] : Exploring smoke: an ethnographic study of air pollution in rural Malawi. *BMJ Glob Health* .6(6) : 10.1136/bmjgh-2021-004970 10.1136/bmjgh-2021-004970 34193474PMC8246283

[2] : Fuel Efficiency and Air Pollutant Concentrations of Wood-Burning Improved Cookstoves in Malawi: Implications for Scaling-up Cookstove Programs. *Energy Sustain Dev* .2017;41: 10.1016/j.esd.2017.08.007 112-120 10.1016/j.esd.2017.08.007 29731584PMC5930929

# Response

**Published:** 2023-02-06

**Authors:** Sepeedeh Saleh

**Affiliations:** Liverpool School of Tropical Medicine, UK

We thank the reviewer for this detailed and insightful review. The following are responses to individual points in the review and we are submitting an edited version of the paper with relevant changes in response to reviewer considerations.

 We are grateful for the observations on our use of mixed methods in exploring air pollution exposures.

 We agree that our experiences and findings support the use ethnographic and survey methodologies in parallel, and we hope to see other researchers follow our lead in this.

 In terms of additional activities which might have contributed to air pollution in and around the village environment, our preliminary ethnographic work (reported separately) revealed that other smoke-producing activities resulted in much lower exposures, and took place more infrequently, than cooking. This statement has now been added to the paper for clarity.

 We would welcome larger, more formalised studies of this, and research involving individuals spending more time away from the village environment, to extend these findings.

 In response to the query about stove types, wood burning cookstoves were, in all cases, basic chitetezo mbaula, as mentioned by the reviewer. This has now been made clear in the paper. These stoves, present in a few households, were later distributed to all households in the village as part of our study, generating further evidence around their impacts on personal exposures.

 We thank the reviewer for the important questions about the statement *“* *Median PM2.5 exposure during these activity periods was 97.9 µg/m3 (IQR: 22.9–482.0) demonstrating a wide range in terms of level of PM2.5”*. Whilst these data were exposures for overall cooking periods, our regression work - presented under the subheading, ‘Cooking Characteristics’ - allowed further exploration of the role of place of cooking (reflecting ventilation factors) and stove and fuel use. We found that cooking in a kitchen with no roof, walled veranda, or outside the household were all significantly associated with higher exposures than cooking indoors. Potential reasons for this – informed also by our extended participant observations – are proposed in the Discussion section.

 We agree with the observations around homogeneity of cooking activities. Although most meals in the village involved nsima (taking between 30 and 45 minutes to cook) and accompanying ndiwo (vegetables, etc, cooked for a shorter period), beans were also cooked at times. We initially attempted to record type of cooking (food cooked) as an additional variable in this study but found it impossible due to complexity involving frequent concurrent cooking of different foods. Our use of individual observations (every 2 minutes) throughout cooking periods, with regression by measurable cooking characteristics, was our attempt at a ‘best estimate’.

 The reviewer also makes interesting observations on the subject of behavioural practices relating to cooking/fuel burning. We selected – on the basis of preliminary observations in the village – the most measurable and likely important characteristics for inclusion in the regression analysis, as mentioned in the discussion. Sadly, individual small-scale actions such as blowing the fire could not be captured in this way, although these were likely to increase individual exposures. Maize cobs and stalks were not burned during the period of observation (this tends to happen mainly during a time-bound period after the harvest) and plastic and other substances were occasionally added to a fire, sometimes to help with lighting, or for disposal, but usually not as a main fuel source. Although there was little active drying of wood fuel, we would have ideally measured the dampness of wood as this can be an important factor. This measurement was outside the scope of our study, however. Factors such as extended cooking times and continuation of stove burning for heat after cooking were implicitly included in our measurements as we categorised all 2-minute observations as ‘cooking’ or ‘non-cooking’ (with cooking periods extending to the point at which the fire was extinguished). In relation to the use of lighting, fires were rarely – if ever – used solely for lighting in this setting (although undoubtably the evening cooking fire also provided light). This has now been clarified in the ‘settings’ section.

 Regarding the use of CO as a proxy for PM2.5, this was seen more commonly in the past, but is reducing in frequency due to the increasing scientific consensus critiquing its validity for use in this capacity, as well as the rise of smaller, more convenient PM2.5 monitors, such as those employed in our study. Thus our findings both added to the literature on the relationship between these two outcomes (in particular finding differences in the strength of correlation by the presence of absence of specific cooking activity in the near vicinity) and provided early evidence of the feasibility of use of these newer portable PM2.5 monitors for conducting personal monitoring. This has been clarified in the text, with references as appropriate.

 We thank the reviewer for noting the issue of seasonal variation in cooking and other practices. Our extended ethnographic work revealed that, whilst diet and main cooking practices in the village varied little throughout the year, there were small differences such as the use of maize cobs/stalks as fuel around harvest time and roasting of a little maize on charcoal for eating around this time also. There was also likely to be seasonal variation in the dampness of wood fuel, depending on the season. These are all elements of ‘seasonal differences’ noted briefly but not discussed in detail in the discussion, due to limitations in space. Further work on these factors would be valuable.

 Finally, we have added the main language used, correctly identified by the reviewer as Chichewa, into the text. Related issues of language are discussed in more detail in the main ethnographic paper.
